# Supplementary material for: HIVprotI: an integrated web based platform for prediction and design of HIV proteins inhibitors
Source: J Cheminform. 2018 Mar 9;10:12. doi: 10.1186/s13321-018-0266-y (PMC5845081; doi:10.1186/s13321-018-0266-y)
Supplement: Supplementary file 2 — Additional file 2. Source code of HIVProtI web server. [file 13321_2018_266_MOESM2_ESM.zip › HIVprotI_Source-code/hivprotI/weka-3-6-6/doc/weka/classifiers/trees/m5/Impurity.html]

Impurity


---


|  |  |  |
| --- | --- | --- |
| **Overview** \| **Package** \| **Class** \| **Tree** \| **Deprecated** \| **Index** \| **Help** \| **Weka's home** | |  |
| **PREV CLASS**   **NEXT CLASS** | **FRAMES**    **NO FRAMES**     **All Classes** |  |
| SUMMARY: NESTED \| FIELD \| CONSTR \| METHOD | DETAIL: FIELD \| CONSTR \| METHOD |  |


---


## weka.classifiers.trees.m5 Class Impurity

```
java.lang.Object
  weka.classifiers.trees.m5.Impurity
```

**All Implemented Interfaces:**: RevisionHandler

---

``` public final class Impurity extends java.lang.Object implements RevisionHandler ```

Class for handling the impurity values when spliting the instances

**Version:**
:   $Revision: 1.8 $

**Author:**
:   Yong Wang (yongwang@cs.waikato.ac.nz)

---

**Constructor Summary**

`Impurity(int partition,
int attribute,
Instances inst,
int k)`
  
          Constructs an Impurity object containing the impurity values of partitioning the instances using an attribute


| **Method Summary** | |
| --- | --- |
| `java.lang.String` | `getRevision()`             Returns the revision string. |
| `void` | `incremental(double value, int type)`             Incrementally computes the impurirty values |
| `java.lang.String` | `toString()`             Converts an Impurity object to a string |

**Methods inherited from class java.lang.Object**

`equals, getClass, hashCode, notify, notifyAll, wait, wait, wait`

**Constructor Detail**

### Impurity

```
public Impurity(int partition,
                int attribute,
                Instances inst,
                int k)
```

:   Constructs an Impurity object containing the impurity values of partitioning the instances using an attribute

    **Parameters:**: `partition` - the index of the last instance in the left subset: `attribute` - the attribute used in partitioning: `inst` - instances: `k` - the order of the impurity; =1, the variance; =2, the stardard deviation; =k, the k-th order root of the variance


**Method Detail**

### toString

```
public final java.lang.String toString()
```

:   Converts an Impurity object to a string

    :   **Overrides:**: `toString` in class `java.lang.Object`
    :   **Returns:**: the converted string

---


### incremental

```
public final void incremental(double value,
                              int type)
```

:   Incrementally computes the impurirty values

    :   **Parameters:**: `value` - the incremental value: `type` - if type=1, value will be added to the left subset; type=-1, to the right subset; type=0, initializes

---


### getRevision

```
public java.lang.String getRevision()
```

:   Returns the revision string.

    :   **Specified by:**: `getRevision` in interface `RevisionHandler`
    :   **Returns:**: the revision


---


|  |  |  |
| --- | --- | --- |
| **Overview** \| **Package** \| **Class** \| **Tree** \| **Deprecated** \| **Index** \| **Help** \| **Weka's home** | |  |
| **PREV CLASS**   **NEXT CLASS** | **FRAMES**    **NO FRAMES**     **All Classes** |  |
| SUMMARY: NESTED \| FIELD \| CONSTR \| METHOD | DETAIL: FIELD \| CONSTR \| METHOD |  |


---
